# Correlation Analysis to Identify the Effective Data in Machine Learning: Prediction of Depressive Disorder and Emotion States

**DOI:** 10.3390/ijerph15122907

**Published:** 2018-12-19

**Authors:** Sunil Kumar, Ilyoung Chong

**Affiliations:** Department of Information and Communications Engineering, Hankuk University of Foreign Studies, Seoul 02450, Korea; sunil75umar@hufs.ac.kr

**Keywords:** correlation analysis, health care, machine learning, data analytics

## Abstract

Correlation analysis is an extensively used technique that identifies interesting relationships in data. These relationships help us realize the relevance of attributes with respect to the target class to be predicted. This study has exploited correlation analysis and machine learning-based approaches to identify relevant attributes in the dataset which have a significant impact on classifying a patient’s mental health status. For mental health situations, correlation analysis has been performed in Weka, which involves a dataset of depressive disorder symptoms and situations based on weather conditions, as well as emotion classification based on physiological sensor readings. Pearson’s product moment correlation and other different classification algorithms have been utilized for this analysis. The results show interesting correlations in weather attributes for bipolar patients, as well as in features extracted from physiological data for emotional states.

## 1. Introduction

**Figure 1:**
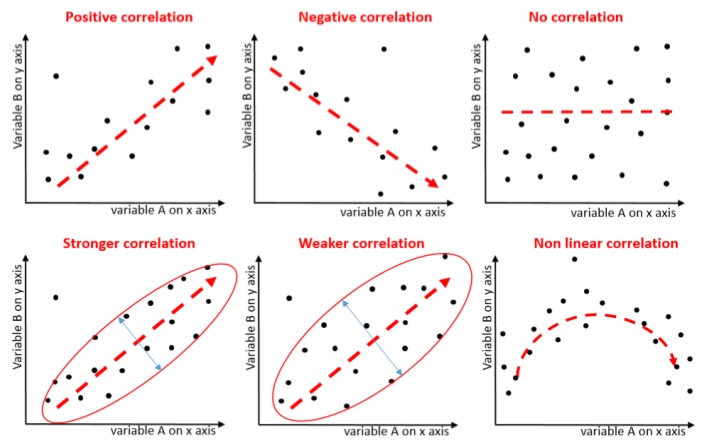
Correlation based on direction, form, and dispersion strength.

In order to analyze the causality in a dataset, various techniques can be used. One such technique in the domain of data analysis is frequent pattern mining. In this case, two types of analysis are widely used: identification of association rules and correlation analysis. In association rule mining, frequent pattern analysis is performed using the support count and confidence measures. An association rule will be considered strong if its support count and confidence are above some defined threshold. However, the identified rules are sometimes irrelevant and can often be misleading. This limitation can be handled by correlation analysis, which is used to determine the strength of a relationship between two item sets [1]. In this case, the strength can be identified based on three criteria: direction, form, and dispersion strength, as shown in Figure 1. The limitation of this analysis is that in the case of two independent variables, it cannot determine the causality between the two. That is, we will not have any idea which variable is affecting and which one is getting affected. Another situation that can cause distort the correlation and association results is the confounding of another attribute [2]. If this attribute is not considered in the dataset, then its effect may not be highlighted in the experimentation and thus leading to spurious analyzed correlations. In this regard, determining predictor attributes plays a very critical role, as we try to include as many relevant attributes as possible, to avoid such circumstances. Correlation analysis is a widely used statistical measure through which different studies have efficiently identified interesting collinear relations among different attributes of datasets. This study has employed correlation analysis to identify such attributes which strongly affect depressive disorder severity and emotional states.

There are several factors that have been found leading to a higher number of depressive disorder cases in South Korea [3,4,5], including work pressure and overtime, loneliness, social relations, socioeconomic status, etc. However, the effect of weather conditions, which may have a considerable impact on depressive disorder, has often been overlooked in this region. With respect to emotion data analysis, the most commonly-used methodologies in practical environments and research approaches involve emotion detection through facial expressions, physiological sensor signals, speech recognition and analysis, and text analysis [6]. The former two techniques are more commonly used, because facial expressions and physiological responses are more closely related to one’s emotional reactions, whereas limited information can be extracted regarding emotional reactions in the latter two techniques [7,8]. Besides, in the latter two approaches, the responses of emotional reactions can be manipulated, hence resulting in more noisy data and obstructing analysis [9,10]. This can also be the case with facial expressions, where we have a lot of noisy data, as compared to physiological responses. In this work, we have considered the first two techniques for identifying a patient’s emotional state.

This work considers aspects of healthcare service provisioning using data analytics. Therefore, different challenges are addressed and overcome in this study. One of the essential problems addressed by this work is the process of identifying information that has a strong impact on depression for specified patients. Considering different studies that have identified different types of depressive disorder situations, each patient shows different behavioral trends based on his or her specific depressive disorder type and its symptoms. As there are different data sources through which we can analyze behavioral trends (facial expressions, physiological sensor signals, speech analysis, text analysis, self-reporting questionnaires, etc.), there is a strong need for mechanisms that identify such parameters with strong relationships specific to certain depressive disorder patients. Another challenge is identifying prominent and persistent relationships over longer periods of time. As there can be different levels of uncertainty in the patterns of acquired data (such as high and low levels of mood in bipolar disorder), longer periods of data are required to analyze the trends. A good prediction model requires enough data readings to acquire sufficiently steady trends to have reliable prediction results. Further, a system is required for on-demand data acquisition from different data sources based on the specific user context. Based on the above discussion, this study aims to achieve the following objectives:

- Analyzing the impact of weather on two types of depressive disorder; Bipolar and Melancholia disorder.
- Analyzing the impact of physiological data on emotions, as well as identifying patient’s health status, using machine learning with strong correlated attributes.
- Experimentation for data acquisition and analysis.
- Achieving higher accuracies from the proposed methodology and discussing the results based on highly correlated data.

In this regard, the data acquisition process for the relevant information is discussed, which involves the periodic collection of multimodal data including local weather data, patient’s emotional states, and depressive disorder severity information. For correlation analysis, two datasets have been considered with different characteristics. One for emotion detection application and the other is for depressive disorder situation analysis based on weather. Based on the identified correlations, strong attributes have been ranked and filtered in order to optimize the performance of the machine learning. Such attributes can be used to further analyze and predict the patient’s health status.

The rest of this paper is organized as follows. Section 2 discusses the related work, which involves studies related to correlation analysis and the approaches that can be used to identify strong predictor attributes. Section 3 describes the correlation and machine learning- based methodology to identify effective datasets for predicting depressive disorder severity and emotional states. Section 4 describes the dataset description used for the data analytics. The experimental use case and results are presented in Section 5. Section 6 discusses the interpretation of results as well as provides limitations and possible direction of this research in the future works. Finally, Section 7 concludes this paper with a summary.

## 2. Related Works 

Various techniques can be used to perform correlation analysis to identify relationships in the dataset. However, their application depends on the type of dataset. Table 1 shows the categorization of common techniques used for correlation analysis based on dataset types. However, each algorithm has its own considerations as well as limitations in identifying the relationships. For example, the Chi square test is used for determining the probability of independence in the distribution, but it does not provide the details of the relationship such as dispersion strength. Other aspects such as assumptions and hypothesis also vary from one algorithm to another. In this work, we have both quantitative and categorical data considering multimodal data. The depressive disorder severities can be represented in % as quantitative values, and can also be defined as none, mild moderate, moderately severe, and severe as categorical values.

Analyzing the effect of one event or occurrence on another is performed in numerous fields of research. Some research on correlation analysis has been undertaken in the financial market, agriculture sciences, imaging data analysis, signal processing, time series data analysis, statistical analysis in social and environmental data, etc. Some examples of financial market analysis include finding the relationship between stock returns in the Central and Eastern European stock market as well as identifying simultaneous trends in the world’s financial markets’ crises [11,12]. Inamdar et al. [13] used Pearson product moment correlation coefficient to identify the errors in hyperspectral image data. In the domain of social web content, the study [14] analyzed the prevalence and impact of different features, such as friends, views, comments, etc. in three popular websites: YouTube, Panoramio, and Epinions. The study shows the application of correlation technique in the time series data analysis [15].

The effect analysis has also been studied with respect to mental health assessment. In this regard, one application of Pearson product moment correlation is presented in emotional analysis using Electroencephalography (EEG) and speech signals [16], which observed high correlation results in the happy and sad emotional states. Huibers et al. [17] performed an association analysis on survey data collected from the south of Netherlands. The main objective of this work is to identify the environmental factors (seasons and weather) affecting mood and depressive disorders using stepwise logistic regression analysis. The environmental parameters consisted of temperature, sunshine, and rainfall, as well as the four seasonal parameters (spring, summer, fall, and winter). The questionnaire was based on the Diagnosis Inventory Depression (DID) for measuring DSM-IV (Diagnostic and Statistical Manual of Mental Disorders) symptoms of depression. The DID-based dataset was comprised of 14,478 responses collected over three years (2005 to 2007). The findings of this analysis did not show any significant effects of environmental factors on mood and depressive disorder. The seasonal changes had effects on depressive disorder and sad mood, with higher rates of both in summer and fall. These results were evaluated through univariate associations of each parameter with depression and sad mood and their odds ratios. Another study [18] that applied stepwise logistic regression studied the relationship between climate changes and depression in different regions of the United States. The data for depression monitoring was acquired from Twitter tweets. Around 600 million tweets were collected from 2013 to 2014, in which the tweets having the keyword “depress” or any of its variations were filtered and selected. Based on the data and the Koppen-Geiger climate classification system, they divided the region into seven climate zones, of which four were selected for analysis. The weather data was obtained from the National Oceanic and Atmospheric Administration (NOAA), involving the records of relative humidity, temperature, sea level pressure, precipitation, snowfall, wind speed, globe solar radiation, and length of day as independent parameters. Based on these parameters, the ratio of total tweets to tweets about depression was evaluated as a dependent variable. The results showed that summer and fall had significant effects on depression rates in all four climate zones. However, the sequence of results in summer was inverse to that of fall. With respect to weather, wind speed, snowfall, and global solar radiation had substantial effects in all zones, whereas relative humidity, precipitation, wind speed, and temperature were effective in specific areas. Sea level pressure was only effective in humid continental areas. Molin et al. [19] used Copenhagen weather data to identify the factors affecting depression. That dataset included temperature, minutes of sunshine, global radiation cloud cover, barometer pressure, precipitation, wind speed, and length of daylight. The symptom data was collected using Beck Depression Inventory (BDI) over a four-year period. The consideration criteria of patients for depressive disorder was at least three episodes in the previous five years. Spearman’s method was used to identify strong relationships among entities, and the least significant parameters were identified and discarded using the backward elimination method for regression analysis. The results based on the Spearman correlation coefficient showed that global radiation, minutes of sunshine, cloud, daylight, and temperature had considerable relationships with BDI. Further, the study found that high temperature and long hours of daylight had an inverse relationship with depression score, hence they were considered to be the best predictors of depression among the parameters. The study carried out by Blanchard et al. [20] also signified another use case of correlation analysis in depressive disorder data. In that study, correlations with posttraumatic stress disorder (PTSD) and symptoms of major depression were analyzed among victims of motor-vehicle accidents. A total of 158 victims were assessed by interviews as well as psychological tests using the Beck Depression Inventory, State-Trait Anxiety Inventory, etc. In the study [20], a statistical software package LISREL (LInear Structural RELations) was used for the analysis and the evaluations scheme involved chi squared and ANOVA tests. The results showed that accident victims can develop PTSD and symptoms of major depression. However, the strength of depression symptoms had no relation with the effect of PTSD. Another study [21] identified the relationships of plasma levels of different vitamins and acids on depression severity using multiple analysis of variance (MANOVA). The results identified significant correlations between glutamate, alanine, and L-serine with patients having severe depression, as assessed by the 21-item Hamilton Rating Scale. Khalili et al. [22] adopted a similar scheme of correlation-based feature selection to improve their prediction results. Their input data included the physiological readings of galvanic skin response (GSR), respiration, blood pressure, and electroencephalography (EEG). Their results showed that the classification algorithm Quadratic Discriminant Analysis (QDA) achieved 1 to 3% increased accuracy by using the feature selection technique. Paul et al. [23] used multi regression model to analysis effect of weather on the patients’ energy levels and sleep. 10 patients were involved in the study in which five were on antidepressant medication. Data regarding health conditions involved behavioral parameters which were evaluated through 1–24 Likert scale, and amount of sleep on daily basis was recorded. The weather data included temperature, sunshine, pressure and relative humidity. The overall results showed that the patients’ mood and energy were highly related with season, as they showed positive mood and high energy in summer and vice versa in winter. Other weather parameters showed varied results based on patients. Besides depressive disorder, effect of weather on emotional states and mood has also been carried out in which Z. Spasova [24] has contributed. This study has evaluated emotional states and mood for 187 volunteers using 4 different psychological tests, involving self-evaluation questionnaire, and one test for the validity for responses. These volunteers were not diagnosed with any severe depressive disorder condition. The weather data involved sixteen weather types. The comparative analysis performed by *t*-test showed that emotional changes are affected by different weather types, however, the favorable weather did not always have positive effect. Besides, they also discussed the effect of abrupt weather changes with negative mood as well as the relationship between psychological behaviors and neuroticism. The study for the effect analysis of anxiety and weather is considered as a research issue. In this regard, Bulbena et al. [25] performed the analysis on emergency reports of a General Hospital in Barcelona for the year 2002. Weather data involved temperature, relative humidity, pressure, wind speed and directions, precipitation, solar radiation and rainfall. Statistical analysis involved Spearman’s correlation and logistic regression. The anxiety episodes were identified as more common with hot wind, rainfall and autumn season. The occurrences of the attacks was reduced significantly on weekends.

## 3. Correlation Analysis Methodology 

### 3.1. Methodology to Identify Strong Predictor Attributes

Correlation analysis determines the strength of a relationship between two item sets, which can be a dependent and an independent variable or even two independent variables [1]. In such a case, the strength can be identified based on direction, form, and dispersion strength, as shown in Figure 1. Numerically, this relationship is usually determined by a decimal value, known as the correlation coefficient. The correlation coefficient is determined under a certain predefined range (depending on the algorithm). Based on the value of the coefficient in the given range, its strength and direction can be determined. The coefficient having positive sign indicates that the two variables are positively correlated, whereas negative sign indicates negative correlation. Higher number of coefficient indicates that the two variables have strong correlation and lower value indicates otherwise. For example, Lift is one correlation measure with a coefficient ranging around one. If the value is greater than one, then the two variables are positively correlated; otherwise, they are negatively correlated. In the case of the value being 1, there is no correlation.

This relationship helps us identify which independent variables can have stronger impacts on the dependent variables, and therefore leads us to more efficiently predict the outcome of a dependent variable. Having a strong relationship, the independent variable can be considered a strong predictor for the dependent variable. The limitation of this analysis is that in the case of two independent variables, it cannot determine the causality between the two. However, in this research, we identify the correlation among independent attributes as well as between dependent and independent attributes in order to identify the strong predictor attributes.

In order to identify the data that have strong impacts on target emotion and depression state, we have adopted Pearson’s product-moment correlation coefficient to study the relationship between each of the independent attributes with target classes for prediction. The product moment correlation was first proposed by Francis Galton in 1880s, then later modified by Karl Pearson in 1896, and has since been known as the Pearson product-moment correlation coefficient. It is a statistical analysis of the collinear relationship between two variables. It involves the ratio of covariance and the standard deviation of the data values between two given variables. Consider two variables A and B. Then the Pearson’s correlation coefficient can be calculated using the following formula:

(1) $$C_{A,B}=\frac{Covariance\left(A,B\right)}{\sigma_{A}\sigma_{B}}$$

where *C_A,B_* is the correlation coefficient, Covariance(A,B) is the covariance, and *σ_A_* and *σ_B_* are the standard deviations of A and B, respectively. In the case of a dataset involving two set, {a_1_, a_2_, …, a_n_} and {b_1_, b_2_, …, b_n_}, the correlation coefficient can be calculated as:

(2) $$C=\frac{\sum_{i=1}^{n}\left(a_{i}-\bar{a}\right)\left(b_{i}-\bar{b}\right)}{\sqrt{\sum_{i=1}^{n}{\left(a_{i}-\bar{a}\right)}^{2}}\sqrt{\sum_{i=1}^{n}{\left(b_{i}-\bar{b}\right)}^{2}}}$$

where *n* is the sample size, $a_{i}$ and $b_{i}$ are the ith data values, and $\bar{a}$, $\bar{b}$ are the mean values. The value of the coefficient (*C*) ranges between −1 and +1. Values close to +1 show strong positive correlation, those close to −1 show strong negative correlation, and those closest to 0 show no relation. Since Pearson’s correlation determine linear relationship, therefore linear form is considered to be analyzed among the variables. For depressive disorder analysis, the independent attributes come from the weather dataset, and for emotion data analysis, the independent parameters come from the physiological signals dataset. In this case, the relationship is identified based on the direction, form, and strength of the dispersion of the data points between the two parameters. However, we cannot determine the strong predictor attributes based only on the correlation coefficient. Further, additional mechanisms are required to identify the combined effect of independent parameters on target classes. Therefore, in order to evaluate the correlation coefficient result, we have used stepwise selection on the applied machine learning models so that we can observe the change in accuracies that should be consistent with the result of the correlation analysis.

**Figure 2:**
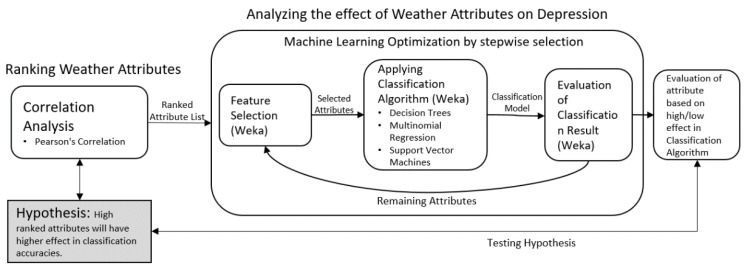
Methodology to identify strong predictor attributes.

Figure 2 shows the steps to be performed. Machine learning and other related tasks have been performed using WEKA tool [26]. In this figure, the hypothesis emphasizes that adding features (weather parameters/physiological signal data) that have high correlation coefficients will have a positive effect on the model’s accuracy, and vice versa. First, we rank the attributes based on the correlation coefficients. Then this result is used in stepwise selection, where different sets of attributes (features) are selected using the feature selection technique. The feature selection technique performs attribute evaluation, which further provides the ranks and their respective weights based on the underlying feature selection technique [27], which in our case is Pearson’s correlation coefficient. Based on the selected attribute set, the machine learning model is trained and evaluated in terms of prediction accuracy. For model testing, 10-fold cross-validation scheme has been adopted. In the datasets involving multiple subjects, LOSO (Leave One Subject Out) is commonly used, however, in this study, we have considered single subject for each case. Therefore, a cross-validation scheme is considered feasible. In order to minimize the possible bias due to division of dataset applied for validation, we have performed 10-fold cross-validation five times with randomly shuffled dataset in each iteration. Finally, the average prediction accuracy is recorded of the five evaluations. This process is in compliance with existing works [28,29,30,31]. Finally, we observe the change in accuracy in order to analyze the combined effect of independent parameters set, based on correlation coefficients. For this purpose, we have used the backward elimination method. This technique is generally considered over forward selection due to some drawbacks of forward selection technique, including the fact that stepwise inclusion of features may result in reducing the significance of any feature already present in the set, i.e., *p*-value > 0.05. Different applications has adopted this method for feature selection [32,33,34].

### 3.2. Correlation-based Attribute Ranking

The Weka platform provides feature selection mechanisms which involve filtration techniques to select relevant parameters in order to optimize the machine learning model’s performance. In this case, the ranking technique assigns weights to the given features based on the evaluation criteria and techniques involved in the model. Then, based on the weights, features are ranked and those features that are most suitable to be applied in the machine learning algorithm, are filtered. There are many different techniques supported in Weka for feature selection, such as Classifier subset evaluation, Chi square attribute evaluation, Relief attribute evaluation, Gain ratio attribute evaluation, etc. In our approach, we have applied Correlation attribute evaluation, in which features are weighted and ranked based on Pearson’s product moment correlation. This technique has been previously described [27]. The main idea behind this approach is that the significance of a relevant feature set in a dataset can be determined by evaluating the correlation of that set with the dependent variable, as well as the correlation among the features. A feature set is considered good for a machine learning model if the features are highly correlated with the dependent class and not correlated with each other. Based on this concept, a feature set can be evaluated as follows:

(3) $$Merit=\frac{\bar{kavg\left(corr_{fc}\right)}}{\sqrt{k+k\left(k-1\right)\bar{avg\left(corr_{ff}\right)}}}$$

where Merit is the correlation between the feature set and the dependent class and is the ranking criteria for evaluating the feature set, *(avg(corr_fc_))* is the average of the correlations between features and the dependent class, *avg(corr_ff_)* is the average of the correlations between the features, and k is the number of features.

Here, *corr* is used in evaluating the feature set in the feature selection algorithm. Therefore, based on the above equation, a feature set can be evaluated based on the following factors:

- Higher correlations among the features indicate lower correlation between the feature set and dependent class.
- Higher correlations between the features and the dependent class indicate higher correlation between the feature set and dependent class.
- Higher number of features indicate higher correlation between the feature set and dependent class.

Based on this ranking technique, feature selection has been performed using Weka.

### 3.3. Prediction based on Ranked Attributes

For training the physiological dataset, involving highly correlated attributes, the Random Forest algorithm has been applied. The Random Forest algorithm [35] uses a bagging approach, unlike the Adaboost algorithm (Adapting boosting). In the bagging approach (Bootstrap AGGregatING), *n* different training samples are defined, and the algorithm is trained on each sample independently. The final algorithm is obtained by averaging all of the prediction results. The training subsets allows for data with replacement. The goal of this approach is to reduce the variance in the result (over fitting). In this case, the dataset is divided into randomly collected samples, and a classifier is applied to each sample. Then, based on voting, the higher occurring prediction output is selected. In Weka, the underlying decision tree algorithm for this algorithm is REPTree. In this case, the tree is generated based on information gain/variance.

Other classification algorithms that we have applied to the Depressive disorder and weather data include: Multinomial Logistic Regression, Logit Boost, Random Forest, and Support Vector Machines. Multinomial Logistic regression [36] is a classification technique that extends the capabilities of logistic regression (handling dichotomous dependent variable) to classifying nominal dependent variables. This is performed by converting a multinomial to a many binomial problem, developing a logistic regression model for each binomial category, then comparing the probability equations of the models. Another approach is to develop k-1 simultaneous models for k classes. The Logit boosting algorithm [37] adopts similar techniques as the Adaboost algorithm (Adapting boosting), by minimizing the logistic loss function of logistic regression in each iteration. Support Vector Machine [38] is another supervised learning algorithm, which creates a model based on a hyper plane. A hyper plane is a plane that best divides the classes of the dataset. An optimal hyper plane is identified by maximum margin, which defines the maximum distance between the nearest data point of each class to the hyper plane. Larger distances lead to more optimal hyper planes. In our analysis, a linear model for the hyper plane has been used in classification.

We have applied different algorithms as it is difficult to select a specific algorithm for model generation, because each algorithm may have different accuracy based on the depressive disorder severity level patterns. These algorithms have been applied without any modification in the core development of the WEKA tool. The algorithms are selected based on the prediction accuracy and compliance of their results after evaluating the models with ranked feature sets. By compliance, we mean that the model’s accuracy is improved after using selected highly ranked feature set (evaluated by ranking algorithm). The prediction accuracy is calculated using following formula:

(4) $$Accuracy=\frac{TP+TN}{TP+FP+FN+TN}$$

where *TP* is True Positive, *TN* is True Negative, *FP* is False Positive and *FN* is False Negative. This accuracy has been evaluated through the confusion metric in Weka. The ranking results by the correlation coefficient are evaluated with these learning algorithms using the stepwise backward elimination technique, and the accuracy for each iteration is compared.

## 4. Dataset Description used for Correlation Analytics

This section describes the relevant datasets which are used for correlation analysis and machine learning. In this work, three types of datasets have been considered:

- Depressive disorder symptom dataset for evaluating depression severity.
- Local weather dataset for classifying depression severity.
- Physiological sensor dataset for emotion detection.

The first two datasets have been used to determine the patient’s depressive disorder situation based on the current weather. The physiological sensor data, on the other hand, is used for identifying the patient’s emotional state. The description involves the metadata for understanding the dataset and data acquisition sources, as well as the preprocessing involved in extracting relevant features, if required for more refined patterns.

### 4.1. Depressive Disorder Symptom Dataset 

There are many different types of depressive disorders, but two of them are considered depressive disorder types which are analyzed in this research: Bipolar depressive disorder and Melancholia depressive disorder [39]. Bipolar is the type of depressive disorder in which the sufferer mostly has high and low mood swings along with similar uncertainty in his energy levels. Any type of depressive disorder can be clinically identified through some predefined symptoms. These symptoms are generally the behavioral, mental, or sometimes physical effects on the patient. For Bipolar disorder, the major symptoms as listed by World Health Organization [40] as well as Smith et al. [41] are: sadness, insomnia (over-sleeping), retardation (slowed movement and working), hopelessness, worthlessness, fatigue or tiredness, elevated feeling and energy for activity, and irritation. Significantly more Bipolar disorder cases have been identified in South Korea than in other countries such as Argentina, Spain, Italy, and US [41]. Melancholia is identified as a severe form of depression where sadness and hopelessness are at their peaks. In addition, this depression has the potential to affect personal lifestyle as well as appetite and sleep [42]. In this case, the major symptoms are sadness, hopelessness, worthlessness, suicidal thoughts and suicide attempts, insomnia, lack of reactivity, loss of interest, weight loss/gain, and retardation. The clinical assessment and evaluation of depressive disorder based on symptoms is mostly done using a self-evaluation questionnaire [17,23,29,43,44,45]. Different studies including the ones discussed in Section 2 have also adopted this technique for obtaining depressive disorder severity information.

In this study, the depressive disorder severity is also obtained from questionnaire responses. Two main data sources are involved in the analysis of depressive disorder: the depressive disorder severity, evaluated from symptom questionnaire responses and the weather data. Two depressive disorder types have been considered in the symptom dataset: Bipolar and Melancholia. Each depressive disorder type is identified by the specified symptoms. Table 2 shows the symptom list for each depressive disorder type. These symptoms are classified as having major or minor impacts in identifying depression severity. The columns “Bipolar Disorder” and “Melancholia Disorder” shows the association of respective symptoms with them. Each symptom has a set of questions to be asked to the teen. This approach has been widely used in clinical analysis in order to identify depression. Table 3 shows the sample questions for symptoms dds.01 (refer to Table 2 for Symptom ID). The number below each response is the response score. The teen may choose any one of the responses: “Yes, always”, “Yes, sometimes” and “No, never”, and the corresponding score of that question will be recorded. Each symptom can have a score in a range of 0 to 100. Based on each symptom score, an aggregated depression score can be obtained. The final score for depressive disorder severity has been labeled as none, mild, moderate, moderately severe, and severe, with ranges of 0–20, 21–40, 41–60, 61–80, and 81–100, respectively. The final depressive disorder severity score has been used as a dependent variable for correlation analysis and training the dataset. One limitation of this study is that the symptom dataset is based on simulated data; that is, the questionnaire responses are generated from simulation, based on which the score is calculated.

### 4.2. Local Weather Dataset 

The weather data consists of weather information for Suwon city in South Korea, from station 471190. This dataset is openly available online [46]. This location has been selected for being the closest to our location of observation whose data is available, at a distance of 20 km approximate. The parameters include temperature in centigrade, atmospheric pressure in hPa, humidity in %, visibility in km, wind speed in km/h, rain, snow, storm, and fog. The air quality data, for the same Suwon city, has been collected from online repository provided by the Korean Ministry of Environment (MOE) and the Korea Environment Cooperation (KECO) [47]. The parameters include Ozone (O_3_), Carbon-monoxide (CO) and Nitrogen-dioxide (NO_2_), all measured in ppm. These two datasets are used for analyzing the effects of weather on depression.

The data considered is for one year of observation from January, 2017 to December, 2017. A total of 182 observations have been recorded throughout the year. The symptom data is collected after every second day and weather data has been considered for the same day. Hence, the datasets are synchronized with respect to dates. Table 4 shows the profile information of the dataset used for the analysis of depressive disorder. Here “Depression Severity” is the score evaluated from symptom questionnaire responses. This is the dependent attribute in this dataset. This dataset is used separately for Bipolar and Melancholia disorder, with their respective depressive disorder scores.

### 4.3. Physiological Sensor Dataset

In order to analyze the effective data in emotional states, the eight-emotion sentics dataset [48] has been adopted for correlation analysis and machine learning. This dataset consists of the physiological response data of a subject with four sensors attached to his body. The physiological signals involve: electromyogram (EMG), blood volume pulse (BVP), galvanic skin response (GSR), and respiration (Resp). The EMG sensor detects the voltage on the skin surface caused by muscle activity. In this dataset, the EMG sensor has been used to detect jaw clenching as a sign to analyze facial expressions and emotion levels at the valence and arousal scale. Its unit of measure is millivolts. A BVP sensor is used to detect heart rate variability through measuring blood volume pulse. It involves a photo sensor, which quantifies the reflected light rays from the skin’s surface. Blood is pumped into the blood vessels after each heartbeat, which results in changes in colors, which then reflect the changes in reflection to the sensor. This sensor has a limitation of precision and is affected by the variability of photo sensor readings. The unit of measure is the percentage of light reflected. GSR is used for quantifying the sweat glands. This signal can be a good source for detecting anger or romantic love, as these emotions can cause skin to sweat, and hence can be identified by GSR sensor. Both the BVP and GSR sensors are attached on the left hand of the subject’s body. The respiration signals are conducted through quantifying inhalation and exhalation based on chest cavity expansion and contraction, respectively. For this purpose, a Hall Effect sensor has been used. This area can monitor steady breathing and can omit gas flow due to other activities such as talking, etc. The unit of measure is the percentage stretch per seconds.

For emotion classifications, eight emotions have been considered, as proposed by Clynes [49]. These emotions and profile information used for their examination has been described in Table 5. The emotional states have been identified and labeled as responses of physiological sensor data when the subject has been shown different images in order to trigger the respective emotional states. For example, for grief, an image of a deformed child or the thought of losing one’s mother was delivered to the subject.

The experiments were performed on one single subject with all four sensors (EMG, BVP, GSR, and Respiration) attached to the subject’s body. The experiment was carried out for an average of five minutes for each emotional state. The frequency of sensor data collection was 20 samples/second. The experiment was performed over 32 days with one 30-min session each day.

#### Feature Extracted from Raw Data 

Since the data collected from physiological signals show sharp peaks and irregular patterns of fluctuations, it is very difficult to find interesting relationships in the raw signal values based on this raw signal data. Therefore feature extraction is a basic requirement for extracting and analyzing meaningful information from raw signals [50]. Therefore, we have extracted six of the total features as described by Picard et al. [51], in order to normalize and smooth the dataset so as to find betters trends and correlations. Table 6 briefly describes each of these features. Feature labels are used as a short form, so that it can be referred in the rest of the paper. These features are extracted for each of the four physiological sensors, therefore a total of 24 features are extracted from the raw dataset. These features have been used in correlation analysis to identify parameters that have strong impacts on detecting emotional states. Table 7 shows the profile information of the dataset used for emotion detection. Attributes are the variables, which include feature extracted from signal data of sensors, EMG, BVP, GSR and Resp, whereas “-f1”, “-f2” etc. are feature labels, described in Table 6. For example, “EMG-f1” is a variable which involves the f1 feature values extracted from raw EMG sensor data. “Emotion” is the dependent attribute in this dataset.

## 5. Experimentation and Results

### 5.1. Experimentation Use Case: Depressive Disorder for Teens

In order to describe the applicability of the data acquisition and analysis based on the defined methodology, a use case has been designed. In this case, two depressive disorder teen-aged patients have been considered, with one suffering from bipolar disorder and the other suffering from Melancholia Disorder. The reason for considering teenagers is the prevalence of depressive disorder and suicide in teenagers [52,53]. There are other symptoms and disorder specifications as well, such as the ones existing in females (postpartum depression, baby blue, etc.) or elderly; however, these are out of the scope of this study.

**Figure 3:**
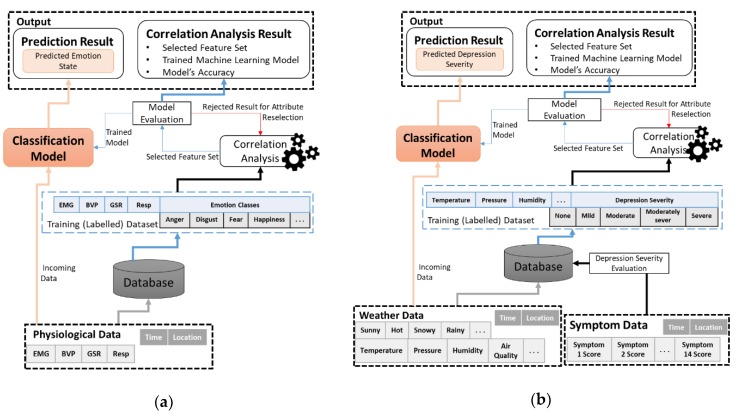
Process flow of Data Analytics: (**a**) Emotion detection. (**b**) Identifying depressive disorder severity.

Approximating depressive disorder situations close to the actual ones requires a high level of information, so that the system’s decisions are justified based on analyzing the different circumstances experienced by the patient. Figure 3 shows the process flow of the use case. We have considered three types of data for the two depressive disorder cases listed above. One is emotion detection through the physiological sensor data. The second is depressive disorder symptom evaluation through the self-reporting questionnaire responses, and the third is weather information based on the patient’s location. The system has three types of interactions with the environment which are related to the three different types of data acquisition. The first interaction is possible via a smartphone application, through which the teen patient provides information regarding his depression symptoms by responding to a questionnaire in the application. Bipolar and Melancholia disorder have their own defined sets of symptoms for evaluating the respective depressive disorder severities, as shown in Table 2. Therefore, each user is given a different questionnaire based on his respective disorder type. These responses are then recorded and stored by the system in the database. In the second interaction, the daily weather status has been acquired based on the teen’s current location. The GPS coordinate system has been used instead of address (city, country etc.) in order to avoid naming errors. The weather information is recorded based on the city or area, covering the specified range. In this case, it is not possible to provide the data of the exact location. Therefore, the weather of the closest proximity has been used. In the third case of physiological signals, the data will be periodically collected from sensors and stored in the database. The eight emotional states have been considered for evaluation, as described in Table 5. All forms of data acquisitions are synchronized with respect to the specified date. This step is crucial for observing the patterns of depression severity, emotional states, and weather information, as well as their effects with respect to date and time. 

The system uses all of the collected information to analyze the health status of the teen patient. This accumulated data is used to highlight the user’s status based on the collective information of weather, emotion, and depression. In this case, the analysis is performed separately for each teen profile and his depressive disorder category. The predicted emotional states are also acquired based on strong effective parameters from the physiological signals. For the machine learning and prediction, the Weka machine learning tool is used in the java platform. To begin, the system retrieves symptom response data with timestamps as the request input. It then performs calculations for depressive disorder severity. It further retrieves emotion data with timestamps as the requested input, then evaluates the depression severity level. For depression severity evaluation based on emotion score, we have referred to the valence and arousal scale described by Scherer et al. [54]. In this scale, anger is labeled as ANGRY, contempt as contemptuous, disgust as disgusted, fear as AFRAID, happiness as HAPPY, sadness as SAD, and surprise as ASTONISHED. From the scale, it can be seen that the “SAD” emotion is closest to “DEPRESSED”. Therefore, it can be considered that users having high scores for sad will also have high chances of being depressed. Hence, the sad emotion score is used to evaluate the depression severity level. Then, it finally retrieves the physiological sensor data with timestamps as the requested input and sends this data to the prediction server in order to obtain the prediction result. For training the prediction model, the model creation server obtains the dataset from the database of a specified time duration. Then, based on that training dataset, it generates a prediction model beforehand. This has been tested using the WEKA tool. The prediction model is then imported in the prediction server, so that it is able to predict based on the incoming requests. The final output of the system is the health status of the particular teen based on: emotional state from Emotion API, predicted state from physiological sensor data, depression severity from questionnaire data, and predicted depression severity from weather information.

This is one component of the Web Objects-based healthcare services provisioning system [55,56]. The proposed system will be used to enhance decision-making capabilities at the Service level, which will further support applications such as forecasting, emergency detection, and notification and recommendation services [57,58,59]. Forecasting will involve future trends in depressive disorder levels and the number of peak levels. Emergency detection will involve suicide detection, triggers that contribute to escalating depression, notifying concerned doctors and relatives, etc. The recommendation service will involve notifying the user, based on his or her preferences [60], with such suggestions that may contribute to minimizing the severity of depression, such as recommending relaxing music, movies, food, etc.

### 5.2. Results of Correlation Analysis

In order to identify the strong predictor attributes for depressive disorder, the Pearson’s correlation-based feature selection technique is used as described in Section 3. Table 8 shows the results of this technique for the depressive disorder symptom dataset. The ranks are ordered from most important to least important attribute for each depression type. Here “Merit” is the weight calculated for each attribute based on the correlation coefficient, which is the ranking criteria described in Equation (3). For bipolar disorder, it can be seen that temperature, atmospheric pressure, season and ozone lie in the top four ranking, and can, therefore, be considered as strong predictors for this disorder severity. The temperature, season and ozone lie in the top four in both depressive disorder categories. In contrast, wind speed, rain, and visibility are evaluated among the lowest ranks in both categories. Storm and Humidity are among the bottom-ranked factors in Bipolar and Melancholia disorder, respectively.

In case of Melancholia disorder, the coefficients’ values are very low, i.e., near zero, therefore showing overall very weak correlation. This can contribute to the fact that the merit is higher than that of the usual case. Hence, it is difficult to determine the effect of weather parameters on Melancholia disorder based on collinear relationship analysis.

**Figure 4:**
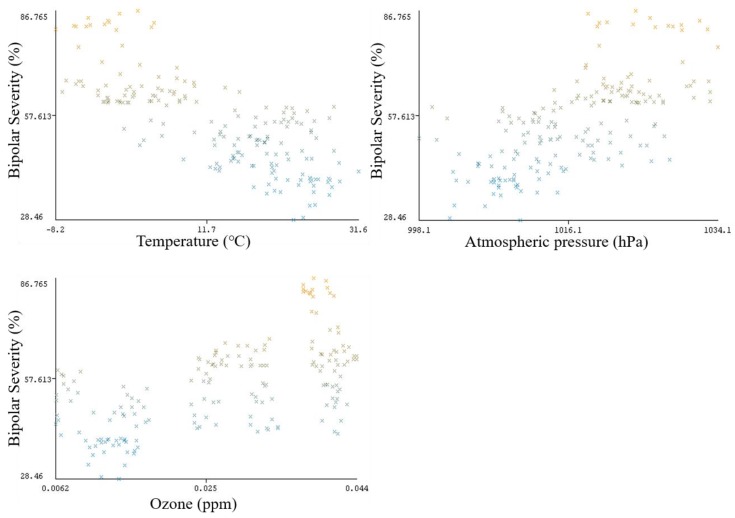
Scatter plot in Weka, of top-ranked weather parameters for Bipolar disorder.

This ranking is an important finding, as it has helped enhance the accuracies of prediction algorithms by applying the stepwise selection technique. This finding has also provided support to the analysis of factors affecting depression. Figure 4 shows the scatter plot in Weka, of the top-ranked weather parameters with bipolar depression severity. The strength of correlation can be related to the results in Table 8. Temperature has the strongest negative correlation with bipolar depression severity, followed by atmospheric pressure, which is positively correlated, and then ozone, which also has a positive correlation with depression.

In order to identify the correlation of each physiological sensor data with emotion, Pearson correlation has been used. Table 9 shows the correlation result and the *p*-value for significance. It can be seen from the result that Pearson’s correlation does not provide any significant result, as all of the correlation coefficients are near zero, and the *p*-value also shows no significance.

Furthermore, we have also analyzed the correlation results among the sensor data with respect to each emotion class. Here, the results also show no or very minimum correlation among the sensor data. Only the readings under “No Emotion” and “Anger” class have small factors of positive correlation between GSR and Respiration sensors, which are 0.325 and 0.456, respectively.

This correlation signifies an inverse effect in analyzing the sensor data attributes. In this case, weaker correlation means that there is less similarity in the patterns of the sensed data. This indicates that each sensor data has unique patterns, which can be an important factor in identifying emotional states. In the case of strong correlation, it would indicate that the strongly-correlated attributes can have the same impact in predicting the emotional states, and hence one of them can be considered a redundant attribute and discarded.

After applying the feature extraction described in Section 3, we applied the correlation over the extracted features and found significant results. Table 10 shows the correlation among the extracted features. Having 24 features makes the correlation table very large, therefore we have selected and shown only the highly-correlated parameters. Here the highest correlations are observed between EMG-f1 and EMG-f2, BVP-f5 and Resp-f5, GSR-f3 and Resp-f3, and GSR-f5 and Resp-f5, each having coefficients around 0.9, followed by correlations between BVP-f5 and GSR-f5 and between BVP-f3 and Resp-f3, each having coefficients around 0.8. In the case of correlation with emotion class, only BVP-f2 showed a small negative coefficient value of -0.38021. Here, we can observe that most of the coefficient values are positive. This is due to two factors: one is that there is no negative signal reading in the dataset, and the second is that very few signal readings show the inverse trend related to the other trend. The difference is only based on the amplitude of the observed signal with respect to the others in the respective emotional state. The ones having negative coefficient values are close to zero. 

The ranking of extracted features has been performed using Weka, based on the approach described in Section 3.2. Table 11 shows the 24 features sorted based on the rank and the respective weight. Here it is observed that f5 and f6 of all four physiological signals are ranked least by the Weka feature selection algorithm. One possible reason for this is that these features involve first and second forward differences of the raw signal readings, which are smaller based on the scale of the values. Therefore, no significant correlation is found in these features.

### 5.3. Machine Learning Results

For predicting the depressive disorder severity levels based on the weather dataset, four classification techniques have been applied as described in Section 3.3. Each of these algorithms has been evaluated with the backward elimination technique in order to analyze the effects of the predictors (the weather attributes) on the dependent variable (the depression severity levels), based on the proposed ranking technique. Each of the classification algorithms has been evaluated with the 10 folds cross-validation scheme in WEKA.

Table 12 shows the classification results for Bipolar disorder, using the backward elimination process. Each row contains the accuracy of each classification algorithm based on the specified number of attributes. These numbers of attributes are decreasing based on the ranking output from Table 8. For example, 13 predictors contain all of the 13 weather parameters used for classification, 12 predictors contain all parameters except for wind speed, 11 predictors also exclude storm, and so on. The other values are the accuracies for each algorithm. Here it can be seen that, as the weak predictors are removed, the overall accuracies of the algorithms increase until around 40–50% of predictor attributes remain. LogitBoost show higher accuracies with up to nine attributes. Therefore, nine parameters from Table 8 are sufficient for predicting depression severity. Support Vector Machine (SVM) shows higher accuracies until four predictors. Random forest shows higher accuracy until eight predictors, and Logistic regression until nine. The highest accuracy in all 44 evaluations is of Random Forest with eight predictors, at 89.01%. Therefore, Random Forest is the most suitable model for prediction in this depression type. From both the observations of feature selection and the classification results, it can be seen that wind speed, storm, visibility, rain, and fog have degraded effects or no effect on training the prediction models. In the case of SVM, its accuracy is highest with only three predictors: temperature, atmospheric pressure, and ozone. With these observations, we have evaluated temperature, atmospheric pressure, and ozone to be the strong predictors for depression severity.

In the case of Melancholia disorder, there is no significant overall change in accuracies, as compared to the results for Bipolar disorder. The main reason for this noncompliance with the correlation result is that the coefficient values are closer to zero than in other depressive disorder types. Therefore, correlation analysis is not effective in this case, and a more complex algorithm is needed to identify relevant relationships.

**Figure 5:**
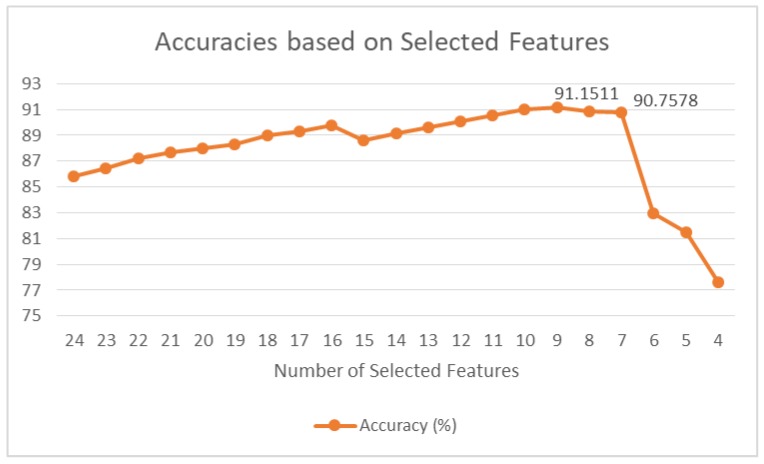
Accuracies of prediction models with respect to stepwise feature selection.

For the prediction of emotional states, we have applied the tree-based approach for the classification of emotions. For this purpose, the Random Forest algorithm has been applied for classification based on the 24 features set. In this regard, the training set consists of 66% of the total dataset, whereas the remaining 34% has been used for evaluations. Figure 5 shows the graph of accuracies with respect to selected features based on the ranking result displayed in Table 11. The features are selected such that the lowest ranked features are eliminated one by one in each step. The first value in Figure 5 is the accuracy of the learning algorithm when all 24 features are selected, and the last value shows the accuracy when the top four features are selected from the ranked list in Table 11. The results show 85.7936% accuracy at 24 features selected for classification. This result is also in compliance with the results in related studies. This learning model is optimized up to 91.1511% with top 9 ranked features. After that, the accuracy reduces as the features with lower ranks are removed.

From these results, it can be concluded that the nine top-ranked features from Table 11 provide the optimum results in the case of using Random Forest as the classification model. In this case, f5 and f6 of all the sensors are eliminated, as they provided lower rankings based on the correlation algorithm.

According to the results shown by Picard et al. [51], the highest accuracy achieved is around 88% using the K Nearest Neighbors (kNN) classifier with only the selected emotional states of Anger, Joy, and Reverence. The accuracy of the trained classifier is much lower in the case considering all emotional states. In our results, the dataset involving all eight emotions has been used; however, only the top-ranked features are selected based on the correlation results. By using a tree-based learning algorithm, Random Forest, the accuracy is improved to 91.511% in our approach. We have also applied the kNN algorithm to train the model at *k* = 10. However, the accuracy is not optimized as well as it takes more time to train and cross-validate the model based on kNN than the Random Forest model.

## 6. Discussions

The results show different characteristics of the dataset as well as different situations are debatable in this aspect. In Bipolar case, temperature, pressure, season and ozone show strong relationship with depression severity. The effect of temperature on depression was also identified by Molin et al. [19]. Whereas the study carried out by Yang et al. [18], identified pressure as effective only in humid areas. Also in other studies, the season was observed as important parameter [17,18], specifically considering peaks of summer and winter. If we consider the machine learning results, when trained and tested excluding wind speed, storm, visibility, rain and fog, showed increase in the accuracies of all the models except SVM. Hence it can be considered that these attributes have least or no relationship with respect to this dataset. In case of rain, storm and fog, one reason for this lack of relationship can be the limited number of observations. Korea is one of those countries where rain is often observed throughout the year, but even then one year is not sufficient for expecting steady trends. Another reason can be the nominal data type. Continuous or ratio based data type may be able to provide more information, for example, mm of rain per minute. In case of Ozone, we cannot be certain of this relationship as the data scatter shows signs of heteroscedasticity. Although, we can apply different tests to identify heteroscedasticity, but still we need larger number of observations for certainty. In case of Melancholia disorder, no significant results were obtained. One of the factor can be the lack of similarity in the dataset as compared to the one acquired from real test subject. Second reason can be the limited number of observations which then fails to provide any steady trends. Third reason can be the limitation of correlation to only collinear relationships. This dataset is required to be analyzed with other techniques to indicate possible relationship with weather. Feature selection technique also plays an important role in filtering out non-relevant features extracted from physiological signals. Using correlation-based feature selection, we observed that f1 and f2 (Table 6) are important features for detecting emotional states. In this study, we have extracted only 6 features, whereas, different other features can also be extracted to analyze new interesting trends, for example, *Z*-score, local maxima, local minima etc. In this study, we have referred to the valence and arousal scale in [54] to highlight the possible relationship of emotion with depression. However, other possible emotional states can also be mapped at symptom level to have more descriptive relationships. For example, “Elevated feelings and energy for activity” (symptom dds.05 in Table 2) can be mapped to “elated”, “EXCITED”, “adventurous” and “aroused” in the valance and arousal scale. In another way, it is also possible to analyze the depressive disorder situation through physiological sensors. In particular, different studies have utilized EEG sensor for analyzing brain signal to analyze depressive disorder situations [61,62,63]. In other case, studies have proved that the stress can be detected using physiological sensors which is an initial state of depression, therefore, it can be easily implied that the monitoring of long term stress using physiological signals can help us in detecting depression [64].

This research has used correlation measure to identify relationships of different parameters affecting mental health situations, specifically the bipolar and melancholia disorder and emotion states. In this regard, there are some limitations that are highlighted with respect to statistical measures, data characteristics and scope. Correlation measure is used to identify the strength of a relationship between two variables. But, it cannot determine the causality between the two variables. In case of independent and dependent variable, this causality covers the aspects of analysis. In case of two independent variables, there can be a possibility of spurious relationship, where the correlation has been produced by extraneous third factor and neither of the variables involved in the correlation has influenced the other. Different tests are used in order to identify such relationship, such as Breusch-Pagan test [65] and White test [66]. In this research, by performing these test we still cannot validate the presence of heteroscedasticity due to limited set of observations. The expected results may also have some uncertain discovered trend or some unidentified relationship due to the limited observations in datasets. As indicated in other studies, the dataset considered usually involves more than one year of observations or higher number of subjects [17,18,19]. Therefore, involving more observations may form steady trends and thus lead to identifying new relationships that are not discovered in the current dataset size. Another point of concern, which requires discussion, is the high correlation values observed in Table 10 (for example: *C* > 0.9). We have considered this problem and have tested PCA (Principal Component Analysis) with physiological dataset using Weka. It was observed that none of the Eigen values were exactly zero, implying that high correlation in the employed dataset does not cause singularity. Nonetheless, similar datasets yielding higher correlation values may cause singularity and result in zero determinant of the feature matrix, which can further lead to issues when modeling different feature selection techniques, such as Principal Component Analysis (PCA). As our future work, we intend to perform comparative analysis amongst various feature selection techniques on physiological dataset to highlight such issues.

## 7. Conclusions

This research aimed to analyze the effects of certain data features on depression severities and the emotional states, in order to predict patient’s current situations. In this case, correlation and machine learning-based data analysis has been performed using different data sources considering specified depressive disorder patients. Based on the correlation and classification results, the parameters that have a strong effect on depression are: temperature, atmospheric pressure, and ozone. In the emotion dataset, the first and second extracted features of all four sensor signals (based on the means and standard deviations of the raw signals) showed a high effect in classifying the emotional states.

Identifying a patient’s health status using data analysis is a very complicated research issue as it involves various aspects, such as work and peer pressure, loneliness and social isolation, conflict in social relations, socioeconomic status, medications, physical impairment and disability, environmental effect, location, incident-based trauma, and so on. This research carries the main assumption which does not consider these situations. In the future, analyzing the impacts of these related factors on our results and also based on real test environment cases will be considered the focal point of the research. Further, longer periods of observation will provide stable and more prominent results in correlation and trend analysis. In this study, a linear correlation model has been used for identifying relationships. However, some weather parameters have complex patterns, so this model could not identify certain relationships. Hence, different correlation models must be analyzed in identifying nonlinear and other relevant relationships in the future.

## Figures and Tables

**Table 1:** Correlation techniques based on data types. The highlighted Spearman’s correlation shows that it considers only ordinal data in the categorical dataset type.

Data Types			Dependent Variables		
			Categorical		Quantitative
			Nominal	Ordinal	
**Independent variables**	**Categorical**	**Nominal**	Chi square test of Independence		Analysis of variance (ANOVA)
		**Ordinal**	Chi square test of Independence	Spearman’s Correlation	
	**Quantitative**		Lift, X^2^ test of Independence (Categorized Quantitative variable)		Pearson product moment correlation, Spearman’s Correlation

**Table 2:** Depressive Disorder Symptoms for Bipolar and Melancholia disorder. The dds keyword in symptom ID (Identifier) represents Depressive Disorder Symptom, followed by the identifier number.

Symptom ID	Symptoms	Bipolar Disorder	Melancholia Disorder
dds.01	Sadness/Worthless/Hopeless	(major)	(major)
dds.02	Insomnia	(major)	(major)
dds.03	Retardation	(major)	(major)
dss.05	Elevated feelings and energy for activity	(major)	
dds.16	Isolation		(minor)
dds.12	Loss of Interest	(minor)	(major)
dds.22	Fatigue	(major)	(major)
dds.10	Anxiety	(minor)	
dds.08	Suicide		(major)
dds.11	Weight Loss/Gain	(minor)	(major)
dds.07	Irritation	(major)	(minor)

**Table 3:** Sample Questions for depressive disorder symptom dds.01. The dds keyword in Question ID (Identifier) represents Depressive Disorder Symptom, followed by the identifier number, followed by q, which represents Question, followed by question number.

Question ID	Question	Response and Score		
dds.01.q1	Do you consider objects and situations as unimportant as you think you are (e.g., homework, grooming, waking up in the morning etc.)?	Yes, always	Yes, sometimes	No, never
		30	15	0
dds.01.q2	Do you feel that you have no value?	Yes, always	Yes, sometimes	No, never
		30	15	0
dds.01.q3	Do you usually walk with you head down?	Yes, always	Yes, sometimes	No, never
		5	2.5	0
dds.01.q4	Do you often have negative statements?	Yes, always	Yes, sometimes	No, never
		5	2.5	0
dds.01.q5	Do you often use gestures that are dramatic and out of context?	Yes, always	Yes, sometimes	No, never
		5	2.5	0
dds.01.q6	Do you feel loss of interest in doing activities?	Yes, always	Yes, sometimes	No, never
		5	2.5	0
dds.01.q7	Do you often perceive your skill set as inadequate for the task at hand?	Yes, always	Yes, sometimes	No, never
		5	2.5	0
dds.01.q8	Do you mostly have negative anticipation about your future?	Yes, always	Yes, sometimes	No, never
		5	2.5	0
dds.01.q9	Do you feel losing affection in things?	Yes, always	Yes, sometimes	No, never
		5	2.5	0
dds.01.q10	Have you ever mentioned some of the following or similar statements: Who could ever want to be my friend? What do my parents really think of me? Why would anyone want to accept the love of a worthless person like me?	Yes, always	Yes, sometimes	No, never
		5	2.5	0

**Table 4:** Profile information of dataset used for depressive disorder analysis. Min Value and Max Value are the Minimum and Maximum values in the dataset. Std. Dev is the standard deviation.

Attribute	Type	Unit	Min Value	Max Value	Mean	Std. Dev
Season	Nominal	-	-	-	-	-
Temperature	Numeric	C	−8.2	31.6	12.748	10.551
Atmospheric Pressure	Numeric	hPa	998.1	1034.1	1016.88	8.083
Humidity	Numeric	%	32	99	64.621	15.665
Visibility	Numeric	km	1.8	20	12.82	5.071
Wind Speed	Numeric	km/h	1.9	15.2	6.481	2.488
Rain	Nominal	-	-	-	-	-
Snow	Nominal	-	-	-	-	-
Storm	Nominal	-	-	-	-	-
Fog	Nominal	-	-	-	-	-
Ozone	Numeric	ppm	0.006	0.044	0.027	0.012
Carbon Monoxide	Numeric	ppm	0.48	0.82	0.64	0.098
Nitrogen dioxide	Numeric	ppm	0.02	0.043	0.033	0.007
Depression Severity	Nominal	-	-	-	-	-

**Table 5:** Description of the eight emotional states examined.

Emotion Class	Source to Trigger	Description	Arousal and Valence Scale
No Emotion	Blank	Boredom	Low arousal and neutral valence
Anger	Images of people arousing anger	Feeling of fighting	Very high arousal and very negative valence
Hate	Image of injustice and cruelty	Anger of lesser severity	Low arousal and negative valence
Grief	Image of deformed child or thought of loss of mother	Sadness	High arousal and negative valence
Platonic Love	Images of family summer	Happiness and peace	Low arousal and positive valence
Romantic Love	Erotic imagery	Lust and feeling for romance	Very high arousal and positive valence
Joy	Song of joy	Stronger feelings of happiness	Medium high arousal and positive valence
Reverence	Images for holly places and reciting prayers	Calm and peaceful feelings	Very low arousal and neutral valence

**Table 6:** Extracted features of raw physiological signals described by Picard et al. [51].

Feature Label	Description
f1	Windowed means of the raw signals.
f2	Standard deviations of the raw signals, based on windowed means.
f3	Windowed means of absolute values of the first forward differences of the raw signals.
f4	Windowed means of absolute values of the first forward differences of the normalized signals.
f5	Windowed means of absolute values of the second forward differences of the raw signals.
f6	Windowed means of absolute values of the second forward differences of the normalized signals.

**Table 7:** Profile information of dataset used for emotion detection. Min Value and Max Value are the Minimum and Maximum values in the dataset. Std. Dev is the standard deviation.

Attribute	Type	Min Value	Max Value	Mean	Std. Dev
EMG-f1	Numeric	1.24	329.11	3.644	6.438
EMG-f2	Numeric	0	192.05	1.147	5.582
EMG-f3	Numeric	0	50.115	0.017	0.196
EMG-f4	Numeric	0	7.29	0.003	0.022
EMG-f5	Numeric	0	50.594	0.014	0.188
EMG-f6	Numeric	0	7.29	0.003	0.021
BVP-f1	Numeric	20.717	58.378	33.545	0.82
BVP-f2	Numeric	1.002	48.439	9.023	4.736
BVP-f3	Numeric	0	36.351	0.085	0.47
BVP-f4	Numeric	0	0.493	0.008	0.022
BVP-f5	Numeric	0	37.159	0.076	0.614
BVP-f6	Numeric	0	0.417	0.007	0.018
GSR-f1	Numeric	1.41	12.996	4.905	2.318
GSR-f2	Numeric	0	0.973	0.025	0.051
GSR-f3	Numeric	0	12.108	0.002	0.071
GSR-f4	Numeric	0	3.358	0.001	0.013
GSR-f5	Numeric	0	12.113	0.002	0.1
GSR-f6	Numeric	0	3.362	0	0.018
Respiration-f1	Numeric	37.853	64.598	56.82	6.802
Respiration-f2	Numeric	0	3.252	0.297	0.319
Respiration-f3	Numeric	0	63.365	0.016	0.707
Respiration-f4	Numeric	0	3.306	0.009	0.023
Respiration-f5	Numeric	0	63.365	0.018	0.999
Respiration-f6	Numeric	0	3.33	0.001	0.016
Emotion	Nominal	-	-	-	-

**Table 8:** Ranking of attributes based on Correlation Coefficient.

Rank	Bipolar-Disorder	Merit	Melancholia-Disorder	Merit
1	Temperature	0.526	Season	243.18
2	Atmospheric Pressure	0.421	Ozone	182.8
3	Season	0.38	Carbon-monoxide	155.3
4	Ozone	0.31	Temperature	102.6
5	Nitrogen-dioxide	0.29	Nitrogen-dioxide	94.4
6	Carbon-monoxide	0.264	Atmospheric Pressure	33.6
7	Snow	0.204	Fog	15.2
8	Humidity	0.179	Snow	10.2
9	Fog	0.13	Storm	6.5
10	Rain	0.125	Humidity	2.43
11	Visibility	0.122	Visibility	1.7
12	Storm	0.105	Wind speed	0.008
13	Wind speed	0.07	Rain	0.003

**Table 9:** Correlation-based ranking for physiological dataset.

Physiological Sensor	Emotion	*p*-Value
EMG	−0.085599801	<0.001
BVP	−0.001507079	0.39
GSR	−0.073850046	<0.001
RESP	−0.023263153	<0.001

**Table 10:** Correlation-based ranking for physiological dataset.

Feature	EMG-f1	EMG-f3	EMG-f5	BVP-f3	BVP-f5	GSR-f3	GSR-f4	GSR-f5	Resp-f3
**EMG-f1**	1								
**EMG-f2**	0.9095								
**EMG-f4**	0.0200	0.6472							
**EMG-f6**	−0.0219	0.2994	0.6227						
**BVP-f5**	4.09 × 10^−5^	0.16253	0.336666	0.647586	1				
**GSR-f3**	0.0037	0.2420	0.2529	0.7875	0.6084	1			
**GSR-f5**	0.0001	0.1733	0.3592	0.5576	0.8610	0.7066	0.3286	1	
**GSR-f6**	0.0002	0.0867	0.1793	0.1491	0.2309	0.333	0.6973	0.4712	
**Resp-f3**	0.0006	0.2431	0.25349	0.86176	0.66583	0.90413	0.23156	0.64011	1
**Resp-f5**	6.76 × 10^−5^	0.17323	0.35939	0.609938	0.941885	0.639795	0.163974	0.905452	0.706954

**Table 11:** Correlation-based ranking for physiological dataset.

Rank	Feature	Weight for Ranking	Rank	Feature	Weight for Ranking
1	BVP-f2	0.12407258	13	BVP-f3	5.1806 × 10^−4^
2	GSR-f1	0.09261232	14	Resp-f5	6.639 × 10^−5^
3	EMG-f1	0.07958459	15	BVP-f5	5.092 × 10^−5^
4	GSR-f2	0.0676282	16	GSR-f5	3.489 × 10^−5^
5	EMG-f2	0.0629491	17	Resp-f3	3.022 × 10^−5^
6	Resp-f2	0.05206328	18	Resp-f6	2.369 × 10^−5^
7	Resp-f1	8.8696 × 10^−3^	19	EMG-f3	2.314 × 10^−5^

**Table 12:** Classification Results for Bipolar Disorder. SVM is abbreviation for Support Vector Machines.

Number of Predictors	Logit Boost (%)	SVM (%)	Random Forest (%)	Logistic Regression (%)
13	85.16	80.77	87.36	79.12
12	85.16	80.77	87.91	80.22
11	86.26	81.32	88.46	83.52
10	86.26	82.42	89.01	84.07
9	86.26	81.32	88.46	85.16
8	85.71	84.07	89.01	84.62
7	85.71	84.62	87.91	84.07
6	84.62	84.62	87.91	81.87
5	85.71	84.62	85.16	84.07
4	82.42	84.62	84.07	81.87
3	74.18	72.53	67.58	72.53
